# Supplementary material for: Roles of Bilirubin in Hemorrhagic Transformation of Different Types and Severity
Source: J Clin Med. 2023 Feb 12;12(4):1471. doi: 10.3390/jcm12041471 (PMC9966404; doi:10.3390/jcm12041471)
Supplement: Supplementary file 1 [file jcm-12-01471-s001.zip › jcm-2169198-supplementary.pdf]

Table S1. Differences of Baseline Characteristics in AIS patients in Two Cohorts.

Variables	sHT corhot (n = 494)	tHT cohort (n = 322)	P-value*
	**Demographic parameters**		
Age (years)	67(58-74.75)	70(61-76.75)	**0.002**
Male n(%)	352(71.3%)	226(70.2%)	0.803
**Vascular risk factors**			
History of atrial fibrillation	66(13.4%)	126(39.1%)	**<0.001**
History of hypertension	302(61.1%)	220(68.3%)	**0.040**
History of Diabetes	130(26.3%)	68(21.1%)	0.108
History of CHD	44(8.9%)	41(12.7%)	0.103
Current smoking	169(34.2%)	125(38.8%)	0.206
Current drinking	147(29.8%)	111(34.5)	0.181
NIHSS on admission, median	7(3-11)	16(11-22)	**<0.001**
mRS on admission, median	2(1-3)	5(3-5)	**<0.001**
	**Biochemistry and vital signs on admission**		
RBC	4.465(4.15-4.78)	4.09(3.65-4.47)	**<0.001**
WBC	7.115(5.735-8.886)	9.635(7.690-11.845)	**<0.001**
Hb	138(127-146)	125(112-138)	**<0.001**
PLT	195(162-230.75)	181.75(149.5-231)	0.074
Glucose	5.4(4.6-6.8)	7(5.8-8.9)	**<0.001**
TBIL	13(9-19)	14(10-19)	0.238
DB	5(3-7)	4(3-7)	0.078
IDB	8(6-12)	9(6-13)	0.132
ALT	19(14-28)	17(12-26)	**0.040**
AST	22(19-32)	24(20-31)	0.857
AKP	83(69-97)	76(63-91)	**<0.001**
γ-GT	34(22-53.25)	31(20.75-51)	0.306
	**Stroke mechanisms**	**<0.001**	
Atherosclerotic, n (%)	430(87%)	147(44.3%)	
Cardioembolic, n (%)	48(9.7%)	141(42.5%)	
Lacunar, n (%)	7(1.4%)	1(0.3%)	
Other causes, n (%)	9(1.8%)	33(20%)	
	**Initial treatment in hospital**		
Anticoagulants	112(22.7%)	159(48%)	**<0.001**
Antiplatelet	356(72.1%)	200(40.5%)	**0.004**

NOTE. HT: hemorrhagic transformation; sHT: spontaneous HT; tHT: HT after thrombectomy; NIHSS, National Institutes of Health Stroke Scale; mRS, modified Rankin Scale; RBC, red blood cell; WBC, white blood cell; Hb, hemoglobin; PLT, platelet; TBIL, total bilirubin; DB, direct bilirubin; IDB, indirect bilirubin; ALT, alanine aminotransferase; AKP, alkline phosphatase; AST, aspartate amino transferase; γ-GT, γ-glutamyltranspeptidase. *Continuous variables were compared between the groups by the Student's t-test or the Mann–Whitney test. The chi-square test was used for categorical variables.

Table S2. Unvariate Analysis of AIS patients in Two Cohorts.

	sHT cohort		tHT cohort	
**Variables**	**OR (95%CI)**	**P-value**	**OR (95%CI)**	**P-value**
TBIL				
Q1	Ref		Ref	
Q2	1.793(1.164-2.762)	**0.026**	1.99(1.170-3.385)	**0.033**
Q3	2.681(1.734-4.145)	**< 0.001**	2.083(1.221-3.551)	**0.024**

Q4	5.128(3.251-8.089)	**< 0.001**	4.430(2.474-7.933)	**< 0.001**
AF	2.050(1.303-3.226)	**0.008**	1.110(0.762-1.615)	0.648
mRS on admission	1.885(1.629-2.181)	**< 0.001**	1.348(1.097-1.656)	**0.017**
NIHSS on admission	1.174(1.133-1.216)	**< 0.001**	1.025(0.997-1.054)	0.137
WBC	1.157(1.077-1.243)	**< 0.001**	1.107(1.045-1.173)	**0.004**
Hb	0.989(0.980-0.999)	0.058	1.009(1.000-1.018)	0.090
Glucose	1.195(1.111-1.282)	**< 0.001**	1.170(1.092-1.253)	**< 0.001**
PLT	0.990(0.986-0.993)	**< 0.001**	0.996(0.993-0.999)	**0.013**
ALT	1.006(0.999-1.013)	0.136	1.009(0.998-1.019)	0.172
AST	1.016(1.004-1.027)	**0.023**	1.010(0.999-1.021)	0.063
γ-GT	1.004(1.000-1.009)	0.062	1.002(0.995-1.009)	0.590
Anticoagulants	1.669(1.166-2.389)	**0.019**	1.077(0.747-1.553)	0.738
Antiplatelet	0.151(0.102-0.224)	**< 0.001**	0.691(0.473-1.010)	0.109

Note: HT: hemorrhagic transformation; sHT: spontaneous HT; tHT, HT afer mechanical thrombectomy; NIHSS, National Institutes of Health Stroke Scale; mRS, modified Rankin Scale; WBC, white blood cell; Hb, hemoglobin; PLT, platelet; TBIL, total bilirubin; ALT, alanine aminotransferase; AST, aspartate amino transferase; γ-GT, γ-glutamyltranspeptidase.
